# Health research and knowledge translation for achieving the sustainable development goals: tackling the hurdles

**DOI:** 10.1093/eurpub/ckaa032

**Published:** 2020-05-11

**Authors:** Karin R Sipido, Iveta Nagyova

**Affiliations:** c1 Department of Cardiovascular Sciences, KU Leuven, Leuven, Belgium; c2 Department of Social and Behavioural Medicine, Faculty of Medicine, PJ Safarik University, Kosice, Slovakia

## Abstract

We are far from reaching the sustainable development goals (SDGs) for health despite a wealth of novel insights in disease mechanisms and possible solutions. Why have we failed in knowledge translation and implementation? Starting from the case of cardiovascular diseases as one of the most prevalent non-communicable diseases, we examine barriers and hurdles, and perspectives for future health research. Health has multiple links with other SDGs. To accelerate the progress towards a healthy society, health research needs to take a broader view and become more cross-disciplinary and cross-sectoral. As one example, behavioural studies will underpin better prevention and treatment adherence. The next generation workforce in health and research needs an adapted education and training to implement more effective health approaches. As well, only effective dialogue and communication between researchers, practitioners, society and policymakers can lead to translation of evidence into policies, addressing the complexity of socioeconomic factors and commercial interests. Within Europe, health research needs a comprehensive vision and strategy that connects to achieving better health, as one of the interconnected SDGs.

## Why has progress towards better health for all slowed down?

Despite a huge increase in medical and public health knowledge, we are still far away from reaching our goals.^1^ Is it because we fail to close the chasm between what we know about health determinants and what we put into practice to improve health? Is it because of a lack of policies to implement knowledge translation? Is it because the targets we are aiming at are wrong? If we want to achieve better health, we have to take a broader view of health research, use novel understandings of knowledge translation and implementation, supported by visionary policies and leadership.^2^

Non-communicable disease (NCD) and chronic disease are one of the major health challenges globally.^3^ Overall, 70% of all deaths are caused by chronic diseases.^4^ They account for 90% of mortality in high-income countries, and more than 75% chronic disease deaths now occur in low- and middle-income countries.^4^ In 2013, premature deaths due to major NCDs (cardiovascular diseases, cancers, respiratory diseases and diabetes) cost EU economies 0.8% of GDP.^5^ Estimates for NCD-related health care costs in the EU include just under €111 billion in 2015 for cardiovascular disease^6^ and €51 billion for cancer in 2009.^7^

Research and clinical experience has reliably and repeatedly documented the role of social, economic, political, environmental and behavioural forces in determining health, disease, treatment outcome and recovery in chronic diseases. These factors are part of overall risk that can be mitigated and illustrate the interaction and relation between several sustainable development goals (SDGs). Consequently, practice and policy needs to take this integrated, transdisciplinary and trans-sectoral view as well. Biomedical, clinical and public health research generate new knowledge, inspire and guide innovation and implementation into practice and public policies. In this simple view of a path to better health, the public and private sectors take up specific roles, with smooth transitions and collaborations. Taking stock of successes and failures of the path from research to health leads to a more complex and richer picture. Advances in health research are the result of interactions between stages of research, relying on a diverse set of actors, engaging in cross-disciplinary research. Engineering and physical sciences have taken their place into biomedical and clinical research for several decades now, with a new wave in the recent era of digitalization and artificial intelligence. Less advanced is the integration of insights from humanities, from psychology and behavioural sciences, social sciences and political sciences. Knowledge implementation to achieve better health requires a next level of activity and is doomed to failure without proper communication between researchers, practitioners, citizens and policymakers.

## Research as an instrument for better health

Does investment in health research pay off? Research to build knowledge is mostly supported through public investment and the return on investment became an important public debate in the late 20th century. The so-called ‘valley-of-death’ referred to a disparate growth of discovery research with more limited growth of novel products and therapies.^8^ Different programmes have since stimulated the so-called translational research, and several measures have encouraged and supported innovation in start-ups and beyond. Health economic analyses in a number of diseases areas have calculated the return on investment in research in hard currency, convincing policymakers of the continued support to research.^9^ Novel therapeutics coming to market, growth of biological therapies, diagnostics to guide more personalized treatments and novel technologies for health monitoring support an optimistic view.

However, on a global scale, we are far from reaching the aims of the SDG on health and well-being. This relates to inequalities in advancement in countries and regions, as well as systemic failures in advancing health. To go into more depth of the role of health research, cardiovascular diseases are a good example in case, because of their prevalence and because there is many data to illustrate the different levels, where research can make a difference and where it so far has worked—or failed. Many of these hurdles are similar across diseases.

## Cardiovascular disease as a success story of research and of health policies—or not?

As a major killer myocardial infarction has been the focus of extensive research. Over the years, this has led to major improvements in outcome.^10^ Breakthrough innovation in the treatment of acute coronary occlusion has led to a dramatic decrease in mortality of acute myocardial infarction: from 13% at the time of introduction of thrombolysis in the ‘80s to 3% and below with the addition of percutaneous intervention and stenting to relieve coronary stenosis. Cardiovascular medicine has championed therapeutic insights and progress through large randomized clinical trials, establishing the efficacy of interventions and drug treatment.^11^ Areas where novel drug development was less successful, such as arrhythmias, benefited from innovative devices for defibrillation and synchronization therapy. ‘Evidence-guided treatment’ is an overarching major topic in cardiovascular publications highlighting the impact of research, clinical trials and registries in cardiovascular medicine.^12^

Extensive population studies, complemented with mechanistic basic research, have identified major risk factors, such as hyperlipidaemia, smoking, alcohol consumption, poor dietary habits and lack of physical activity.^13^ Despite some controversies, the benefits for population health of lowering cholesterol is now well-established.^14^ Basic research and translational research were at the basis of successive drug innovations, with the most recent the PCSK9 inhibitors that have added a powerful biological tool to the therapeutic resources.^15^ The identification of smoking as a major risk factor, shared with respiratory diseases, engendered a large public debate calling for political actions. The resultant policies on smoking, banning smoking in public in many countries within the EU, has had clear and measurable effects on cardiovascular events and health.^16^

These successes of cardiovascular medicine and research have led to false optimism that it is one area where SDG of better health was reached. This view may be an important factor for a lower ranking of cardiovascular research output, measured as publication output, and a stagnation in novel cardiovascular drug development as compared to other medical needs.^17^ The sobering reality is that as a chronic ailment, cardiovascular disease is and will remain a leading cause of suffering, morbidity and mortality, with substantial health care cost in the years to come. This realization is at the basis for calls for action in the USA and in the EU.^18^^,^^19^

Another important reality is that opposed to the aims of the SDGs, many have been left behind. The inequalities in cardiovascular outcomes within the EU are alarming and unacceptable with a two-fold higher mortality in some countries in Eastern Europe compared with North/West Europe.^20^ Cardiovascular disease is an example within SDG ‘Good health and well-being’, but also illustrates the connection between many SDGs and the importance of concerted action. For example, the role of air pollution is well known and is connected to SDGs of climate, clean energy and sustainable cities.

Factors where the system has failed to reach the SDG goal in cardiovascular health are common to many disease areas. Proven approaches, whether in treatment or prevention fail to be implemented, whether for lack of knowledge, of tools or of financial means. Areas of need have no proven treatment. Data are lacking to inform on impact and progress, and thereby allow corrections. The broader context of cardiovascular disease, as for many NCDs, includes social and commercial determinants of heath that are often studied in silos, and require cross-sectoral interventions and health-in-all-policies approach. When looking at these hurdles and at how to tackle them, research is part of the solution. Eventually, reaching the SGDs requires policies and implementation, with measures that transcend the health sector, and with research coming from socioeconomic, political and health systems contexts underpinning such policies. Better communication and an effective research design to produce convincing evidence will empower the necessary political decisions.

## Future sight: tackling hurdles for better health through research

### Enhancing knowledge translation into health practice

Once measures and treatments have been identified, why are they not adopted? For chronic disease, we know the importance of unhealthy behaviours. Eliminating those risks would make it possible to drastically reduce heart disease, stroke, type 2 diabetes and cancers. Adverse psychosocial influences, including negative outlook, social isolation, depression and work stress, have just as negative effects on health, and they act separately from the harm produced by unhealthy behaviours.^21^^,^^22^ We know that people get sicker or fail to recover because they do not adhere to treatment regimens.^23^ Constructing a bridge across health care’s translational chasm requires behaviour change.^24^ We need further investment in studies of how to modify adverse behaviour and evaluate the efficacy of intervention.^25^ Examples include weight management; optimizing health behaviours among older adults; behavioural health involvement in the patient-centred health home. Bottom-up initiatives, where families and communities take charge of their health, can be very successful.^26^ Innovative approaches should engage young children into healthy lifestyles, and new knowledge highlights the benefit from programmes that target mother and child.^27^ Patients’ adherence to treatment or adoption of a healthier lifestyle are areas where innovative support devices and apps form a growing and competitive market. Establishing the value and potential benefit of such devices however requires research with rigorous evaluation.^28^

Health care professionals have to adopt different behaviours as well. A scarcity of crosstalk between practitioners and researchers has long been discussed. One consequence of this disconnect is that limited research knowledge, including implementation of prevention, is adopted into practice.^29^ Implementation by the medical profession of new concepts and treatments remains in the sphere of guidelines, not law, and therefore dependent on willingness and commitment, helped by peer pressure and ethical norms. Behavioural studies have identified professional attitude resisting change but addressing the causes and change management are still in early days, asking for further study.

Overall, there needs to be more attention on behavioural sciences in designing implementation of health measures—including health care professionals and citizens across their life span.

### Translating knowledge into health policies

Although there is many data to inform policies, translation into policies and implementation into public health measures remains the Achilles heel for better health. Lack of efficient communication between researchers, health care practitioners and decision makers is one of the hurdles.^30^ Improved cross-talk might nudge payers towards more rational, less fragmented coverage of better quality care. More strategic alignment of incentives and smoother integration of public health and clinical preventive efforts could yield a lot more population health.

Short timelines of political mandates and lack of political willingness to impose regulations are another hurdle preventing the translation of knowledge into action.^31^ These are challenges for national health systems and are root causes of health inequalities between countries, as well as within countries. Political action should not be limited to the health ministries. Incisive knowledge on social determinants of health, on the role of education, living, working and housing conditions, is available but interventions are not sufficient to implement health equity.^32^

A major challenge is the collusion of health policies with commercial interests, often presented as wider economic interests, e.g. employment. In preventing NCDs, regulation of food composition and labelling, nudging towards healthy diets in schools, alcohol and tobacco (and soon vaping) measures, have a strong knowledge basis but are stopped short of full implementation in the face of industry barriers.^33^

The major gains in health combating infectious disease through vaccination are under threat because of vaccination hesitancy. Tackling this crisis will require a global and cross-sectoral action.^34^

Impact assessment of policies through monitoring outcomes with appropriate standards, registries and exchange of information, will give support to policies and is a potential way forward to address inequalities.^35^

Health research itself needs a proper regulatory framework that facilitates evidence-building, data sharing and knowledge translation.^36^ Current legislation is complex with divided competences between European and national governments, who must provide comprehensive guidelines.^37^

### Fostering a next generation of health researchers and implementers

To move the SDGs forward through research requires a well-trained workforce. Different disciplines have to work together, adopting a common language for communication and new tools for collaboration.^38^ We need further investment in educating the interprofessional workforce about evidence-based practice, and the science and practice of teams. New skills need to be included in the curriculum. Personalized medicine is often viewed as data-driven, requiring training and collaboration in digital data handling. But, above all, ‘personalized’ medicine should be person-driven and implies learning a patient-centred approach.^39^ Health informatics has changed and will continue to change the way we communicate, practice and study health and illness.^40^ Understanding health economics and its place in policies should be part of the skills and competences of a research team that aims for better health. Cross-sectoral training eases the way to innovation.

The presence of well-trained health professionals is essential for health and access to care. Emigration of health care workers is one of many factors contributing to health inequalities in Europe.^41^ The health research workforce is equally important. Mobility for training from low-income to high-income countries must be complemented with reverse mobility for capacity building.

Designing health research for impact and implementation, means thinking ahead and incorporating a vision towards implementation from the beginning.^42^ Citizens and patients as major stakeholders are participants in design, execution and implementation.^43^ Taking up their role, they are a driving force in multi-stakeholder initiatives and training programmes (EUPATI https://www.eupati.eu/). Knowledge brokering and translating science-based evidence into achievable political goals, need training in communication of researchers and health professionals as well, for a fruitful dialogue between all actors.^44^

### Growing relevant knowledge

Despite the major progress that can be made through implementation of relevant knowledge, we should not abandon discovery research. Cardiovascular diseases are but one example of unmet medical needs as targets are changing with time: from treating an acute event to support the failing heart in chronic disease. With better cancer survivorship, new challenges emerge, as e.g. a higher incidence of heart disease due to cardiotoxicity of anti-cancer drugs. In the area of prevention and health promotion, knowledge is still fragmentary and again needs to adapt to a changing world. The recognition of the interrelation of health with other SDGs such as climate, environment, education, animal health and others, implies that research in these areas should be part of an integrated health research vision. It calls for an increased research effort using novel tools and technological opportunities, in interdisciplinary teams. Sustained, and where necessary increased, strategic investment should open up novel research areas, addressing health needs, with people and patients at the centre. The fast development of preventive and therapeutic interventions, of disruptive innovation in data handling and automated analysis, need an intensified dialogue and ethical reflection.^45^

Research on the design and efficacy of health policies and interventions, e.g. addressing environmental and social determinants, is essential.^46^ Understanding and research into the hurdles for knowledge brokering and translation into policies should be part of necessary implementation research.^47^ Health measures need monitoring and research to evaluate the effects, and provide corrective action as needed. This implies a design where stakeholders, policymakers, citizens and patients, are involved from the beginning. For a fast track to innovation and for support of health policies, research data must be of the highest quality. Concerns about lack of proper translational standards in discovery research, questionable integrity of data and sloppy science, are hurdles that are of particular concern in health research.^48^^,^^49^ Measures include a ‘policing’ of publications, but especially encourage a more constructive approach emphasizing the rewards of impactful research through shared data and open science, which comes with an internal quality control. As health research becomes more interdisciplinary, exchange and agreement on methodology and shared standards should ensure quality data.

Finally, ‘research on research’, such as examining health research practice and evaluation of societal impact, can support the health field to improve data quality and to provide strategic data for efficient interactions with politicians and society.^50^^,^^51^ Recent work on the taking up of scientific publications by citizens points out the risks for bias in communication, another area for research.^52^

## A long-term vision and strategy for health and health research

Achieving better health requires leadership that transcends borders. Under the EU treaty, direct competence for health may be limited but there are many channels and instruments to influence health policies directly or indirectly.^53^ As one example, the EU competence in research and innovation is a powerful tool to provide the necessary data and evidence for better health and health care as well as for policies.

Fragmentation at EU level is a cause for concern. Investment in health research will benefit from a cohesive European-wide agenda, and a vision and leadership that can connect Europe and the world.^36^ The next EU research framework programme Horizon Europe promises a joint steering of the cluster Health between DG SANTE and DG RTD. It would be a big step forward. Yet what is missing is a platform for science-led input that connects stakeholders involved in health research and can provide true co-creation of a people-centred and inclusive vision and strategy. A research strategy must be able to adjust to shifting targets and requires a continuity in leadership.

The cooperation of EU and national governments in developing a strategic agenda is essential. Instruments such as structural funds can be successfully deployed in health research an implementation to address inequalities.^54^ They will be most effective in a global strategy and joint targets, when provided with proper analysis of outcomes and efficiency, and of capacity building.^55^

## Conclusions

Health research has been instrumental for better health and will be one of the instruments to tackle the hurdles that slow down progress of the SDGs. To be effective, health research must be more inclusive and partner with social, behavioural, economic and political research to promote knowledge translation and implementation. We must intensify cross-disciplinary studies and become better communicators with policymakers. At EU level, health research needs a comprehensive vision and strategy that connects to achieving better health. Smart and innovative investment will pay off. Better health is deeply connected to many of the SDGs, such as climate, environment and education, and action in related SDGs will lead to co-benefits for health. As well, better health will benefit progress in other SDGs such as work, economic growth and reduced poverty and inequalities. Health research can thus be part of an interactive cycle of progress towards the SDGs.

**Key points**

- To accelerate the progress towards a healthy society, health research needs to take a broader view and become more cross-disciplinary and cross-sectoral.
- We must invest in better translation and implementation of existing knowledge. Behaviour change studies will underpin better prevention and treatment adherence.
- Putting in place facilitating health research regulations and health policies requires an effective dialogue and communication between researchers, practitioners, society and policymakers.
- We must provide proper education and training for the next generation workforce in health and research.
- At EU level, health research needs a comprehensive vision and strategy that connects to achieving better health.
