# Erratum to: Health research and knowledge translation for achieving the sustainable development goals: tackling the hurdles

**DOI:** 10.1093/eurpub/ckaa105

**Published:** 2020-06-08

**Health research and knowledge translation for achieving the sustainable development goals: tackling the hurdles**

This is a correction notice for article ckaa032 (https://doi.org/10.1093/eurpub/ckaa032), published on 23 March 2020.

Some final author corrections to the publisher’s proof had not been implemented. The changes in wording are of stylistic nature and do not affect the content of the article. The corrected version of the paper is now available here (doi.org/10.1093/eurpub/ckaa032). The Publisher apologizes for this error.

